# Deciphering the Proteomic Landscape of Circulating Extracellular Vesicles in Human Abdominal Aortic Aneurysm

**DOI:** 10.1111/jcmm.70725

**Published:** 2025-08-06

**Authors:** Chaoyang Yu, Ge Zhang, Shaotong Pei, Yifei Zhang, Peiyu Yuan, Renying Miao, Kaisaierjiang Kadier, Pengpeng Zhang, Tianshu Gu, Ruhao Wu, Haonan Zhang, Shiqian Zhang, Bo Yang, Han Wu, Yudi Xu, Ke Hu, Qingfei Xu, Yaxin Chen, Jinliang Wang, Zongao Cai, Junnan Tang, Teng Li, Yan Song

**Affiliations:** ^1^ Department of Vascular and Endovascular Surgery First Affiliated Hospital of Zhengzhou University Zhengzhou Henan China; ^2^ Department of Cardiology First Affiliated Hospital of Zhengzhou University Zhengzhou Henan China; ^3^ Tianjian Laboratory of Advanced Biomedical Sciences Academy of Medical Sciences, Zhengzhou University Zhengzhou Henan China; ^4^ Department of Cardiovascular Surgery The Second Clinical Medical College, Jinan University (Shenzhen People's Hospital) Shenzhen China; ^5^ Department of Cardiology First Affiliated Hospital of Xinjiang Medical University Ürümqi China; ^6^ Department of Lung Cancer Tianjin Lung Cancer Center, National Clinical Research Center for Cancer, Key Laboratory of Cancer Prevention and Therapy, Tianjin's Clinical Research Center for Cancer, Tianjin Medical University Cancer Institute and Hospital Tianjin China; ^7^ Tianjin Key Laboratory of Ionic‐Molecular Function of Cardiovascular Disease, Department of Cardiology Tianjin Institute of Cardiology, The Second Hospital of Tianjin Medical University Tianjin China; ^8^ Department of Respiratory and Critical Care Medicine The First Affiliated Hospital of Zhengzhou University Zhengzhou China; ^9^ Department of Thyroid Surgery The First Affiliated Hospital of Zhengzhou University Zhengzhou China; ^10^ Department of Colorectal Surgery The First Affiliated Hospital of Zhengzhou University Zhengzhou China; ^11^ Department of Oncology The First Affiliated Hospital of Zhengzhou University Zhengzhou China; ^12^ Department of Neurology The First Affiliated Hospital of Zhengzhou University Zhengzhou China

**Keywords:** abdominal aortic aneurysm, circulating extracellular vesicle, noninvasive diagnosis, proteomics, systems biology

## Abstract

Abdominal aortic aneurysm (AAA) is the most prevalent and lethal form of arterial aneurysm, frequently manifesting asymptomatically until a catastrophic rupture occurs. While various diagnostic imaging tools and several potential biomarkers have been explored for the purpose of early AAA screening, the usage of liquid biopsy such as extracellular vesicles (EVs)‐carried protein for the early diagnosis of AAA is still being overlooked. In this study, we enrolled 18 AAA patients and nine healthy normal controls, including data from the National Drug Clinical Trial Organisation—Vascular Surgery (NDCTO) (in‐house cohort) and the Second Clinical Medical College, Jinan University (Shenzhen People's Hospital) (external cohort). We employed Olink's proximity extension assay (PEA) technology based on the plasma EV proteins and first comprehensively characterised the proteomics landscape in circulating EV underlying AAA disease development. A complex profile of differential EV proteins and EV protein–protein interactions network in AAA patients was identified. The differentially expressed EV proteins in AAA patients were found to be significantly associated with several enriched pathways, including the cellular response to cytokine stimuli, inflammatory response, and the regulation of the glucocorticoid receptor (GR) pathway. Moreover, five hub proteins were identified as being of particular significance: these were Interleukin‐4, Interleukin‐6, MCP‐1, Neurturin, and Oncostatin‐M. The Olink proteomics technique was utilised in order to identify these proteins. The significance of these proteins was further validated through Western blotting and enzyme‐linked immunosorbent assay (ELISA) in the external cohort. The five EV proteins displayed reliable performance and robustness for distinguishing AAA from healthy people, revealing high accuracy with AUC values of 0.760, 0.840, 0.800, 0.840, and 0.900, respectively. The present study has revealed the plasma EV proteins landscape within AAA and further uncovered their potential roles in the pathogenesis of the disease. This presents a new direction for clinical diagnosis and management of AAA. Consequently, these five EV proteins have the potential to serve as useful biomarkers for the diagnosis and prediction of AAA. Further research is warranted to explore their potential as therapeutic targets.

## 1 Introduction

An aneurysm is defined as a dilation or bulge in the infrarenal section of the aorta, with a diameter at least 1.5 times that of a healthy aorta or exceeding 30 mm. Such an aneurysm is classified as an abdominal aortic aneurysm (AAA) [1]. AAA is the most common type of life‐threatening aneurysm. The incidence of AAA is approximately five times higher in men than in women. In certain countries, the prevalence among individuals over 60 years of age ranges from 2% to 5%. The weakened structure of the dilated aorta often leads to spontaneous rupture, resulting in rapid death [2]. Epidemiological studies have shown that the prevalence in men over 65 years is 1%–2%, and globally, 150,000–200,000 people die annually from AAA rupture [3]. The prevalence of AAA is closely associated with risk factors such as age, gender, and smoking [4, 5]. The primary cause of AAA is degenerative changes in the arterial wall or other non‐specific factors, rather than atherosclerosis. Indeed, over 90% of cases of AAA are attributable to these degenerative changes. Recent studies have identified a multifactorial pathophysiology for AAA, involving inflammatory responses, activation of matrix metalloproteinases (MMPs), oxidative stress, intraluminal thrombus formation, smooth muscle apoptosis, and degradation of the extracellular matrix (ECM) [6, 7, 8, 9].

At present, ultrasound is a suitable method for AAA screening, while CTA is preferred for the assessment and planning of treatment. However, ultrasound has limitations in patients with complex anatomy, and although CTA and MRA are accurate, they are costly and involve radiation or contrast agent risks. Imaging diagnostics for AAA are usually applied only after significant clinical symptoms appear, by which time irreversible changes in the aorta have occurred, greatly increasing the risk of rupture and death [10]. Consequently, there is a need for an improved risk model, where circulating biomarkers could provide valuable information.

The expression or circulating levels of various proteins have been demonstrated to be associated with the development and rupture risk of aneurysm. For instance, the levels of MMP‐2 and MMP‐9 are significantly elevated in patients with AAA, particularly MMP‐9, which has been shown to degrade collagen and elastin in the ECM, leading to arterial wall weakening and the promotion of aneurysm formation. Consequently, elevated MMP expression signifies ongoing degradation of the arterial wall [11]. Previous research has also shown that CRP induces nitric oxide production, regulates fibrinolysis, and alters the expression of inflammatory molecules and complement, processes directly related to AAA formation [12]. Additionally, Cystatin C, a potent cysteine protease inhibitor, may slow the progression of AAA by inhibiting protease activity, thus preventing the continuous degradation of the ECM and the weakening of the arterial wall [13]. While circulating proteins offer potential for early AAA diagnosis, there are limitations. The majority of protein biomarkers demonstrate only a weak correlation with AAA progression and thus possess limited potential for clinical application.

Extracellular vesicles (EVs) are small vesicles secreted by cells, with diameters ranging from 30 to 150 nm, and contain biomolecules such as proteins, lipids, DNA, mRNA, and miRNA [14]. In recent years, the field of EVs research has attracted significant attention due to the potential of these vesicles as biomarkers, offering the possibility of earlier diagnosis of various diseases [15]. EVs are secreted by specific cell types and carry molecules that reflect the state of these cells, making them capable of earlier and more accurate detection of pathological changes in the arterial wall compared to traditional circulating biomarkers like MMPs or CRP, which lack such cell or tissue specificity [16]. The phospholipid bilayer that encapsulates EVs protects their contents from degradation, providing stability in bodily fluids such as blood, urine, and saliva, and enabling non‐invasive detection. This stability enhances the reliability of detecting EV‐based protein biomarkers, reducing false negatives and increasing sensitivity in early screening. Non‐invasive detection also makes EV biomarkers highly feasible for large‐scale population screening, especially for monitoring high‐risk individuals or early disease detection. Previous studies have shown that EV protein biomarkers derived from endothelial cells and platelets also show potential in the early diagnosis of coronary artery disease [17, 18], complementing traditional myocardial injury markers such as troponin [19, 20]. These EV biomarkers could aid in early myocardial infarction diagnosis and assessment of injury severity [21, 22, 23]. Olink's proximity extension assay (PEA) technology, known for its high sensitivity, specificity, and minimal sample requirements, is suitable for studying various disease mechanisms and clinical diagnostics in personalised medicine [24, 25]. Although advances have been made in circulating EV proteomics for diagnosis, no studies have yet explored its potential for early AAA diagnosis.

Thus, we utilised Olink's PEA technology to profile the proteomic expression of circulating EVs in AAA patients. Through comparative analysis, we identified differentially expressed proteins in circulating EVs. Bioinformatics techniques were employed for target gene prediction and functional enrichment analysis, aiming to uncover potential pathological changes in AAA. This study seeks to identify novel biomarkers, their associated biological functions and pathways in AAA, and potential therapeutic targets.

## 2 Materials and Methods

### 2.1 Participants

This study was conducted in accordance with the ethical principles of the Declaration of Helsinki and was approved by the Institutional Review Board of the First Affiliated Hospital of Zhengzhou University (Approval Number: 2024‐KY‐0095) and the Institutional Review Board of the Second Clinical Medical College of Jinan University (Approval Number: LL‐ZLJS‐2022008). Written informed consent was obtained from all participants prior to specimen collection and subsequent analysis. A total of ten AAA patients and five healthy normal controls were collected from the First Affiliated Hospital of Zhengzhou University as an internal cohort for the analysis of the proteomic expression of circulating EVs in AAA patients by using Olink's PEA technology. In addition, eight AAA patients and four healthy normal controls were collected from Shenzhen People's Hospital as an independent external cohort for the external validation of EV‐carried hub proteins. The diagnosis of AAA was made according to the criteria established by the Society for Vascular Surgery (SVS) and the European Society for Vascular Surgery (ESVS). The diameter of the AAA was measured following the methods recommended by the ESVS [26, 27].

The inclusion criteria for AAA patients were as follows: (1) diagnosis confirmed according to the 2019 guidelines of the ESVS; (2) biological age > 55 years; and (3) signed informed consent for the storage and analysis of blood samples. The exclusion criteria included: (1) evidence of active infection; (2) chronic liver disease (Child‐Pugh score ≥ B); (3) end‐stage renal disease (CKD stage 5 or creatinine ≥ 2 mg/dL); (4) chronic inflammatory diseases; (5) BMI < 20 or > 35; (6) major surgery or illness within the last 30 days; (7) use of immunosuppressive or steroid medications; (8) history of organ transplantation; and (9) pregnancy or lactation.

### 2.2 Plasma Sample Collection

This study recruited patients diagnosed with AAA as well as healthy volunteers. Venous blood samples were collected in Na‐EDTA tubes to prevent coagulation. The blood samples were centrifuged at 1500 *g* for 20 min at 4°C to remove cellular components. The supernatant was then collected and centrifuged again at 3000 *g* for 15 min at 4°C. The resulting supernatant, which is the plasma, was collected, and the first 2 mL of plasma from each sample was rapidly frozen at −80°C to preserve the stability and integrity of the plasma biomolecules.

### 2.3 EVs Isolation

EVs extraction was performed using the ExoQuick reagent (EXOQ5A‐1; System Biosciences, USA) to isolate EVs from plasma, following the manufacturer's protocol. First, 250 μL of plasma was mixed with 36 μL of ExoQuick EVs precipitation solution and incubated at 4°C for 30 min. The mixture was then centrifuged at 1500 *g* for 30 min. After discarding the supernatant, the sample was centrifuged again at 1500 *g* for 5 min. The resulting EVs' pellet was resuspended in 100 μL of sterile phosphate‐buffered saline (PBS) and stored at −80°C for subsequent analysis.

### 2.4 Transmission Electron Microscopy

To ensure the integrity and specificity of isolated EVs, we performed standard characterisation using multiple orthogonal methods. The morphology of the isolated EVs was examined using transmission electron microscopy (TEM). First, 10 μL of the EVs solution was placed onto a copper grid and incubated at room temperature for 10 min. The grid was then washed with sterile distilled water, and excess liquid was removed using blotting paper. Next, 10 μL of 2% uranyl acetate was applied to the grid for negative staining for 1 min, and the excess stain was removed with filter paper. The grid was then air‐dried under an incandescent lamp for 2 min. Finally, the copper grid was observed and imaged under the TEM. TEM confirmed the typical cup‐shaped morphology.

### 2.5 Flow Nanoanalyzer

The concentration and diameter distribution of the extracted EVs were measured using a Flow NanoAnalyzer (FL Sciences).

### 2.6 Western Blot

The protein concentration of the extracted EVs was determined using a BCA Protein Assay Kit. Based on the quantification results, the EVs sample amount (10–30 μg) was calculated. The appropriate volume of 5× SDS buffer was added to the sample, followed by vortex mixing. The mixture was then denatured by heating in a 95°C water bath for 5 min and subjected to protein electrophoresis.

After electrophoresis, the separation gel was removed, and the target proteins within the gel, including EVs‐positive markers (Tsg101 [#ab125011, Abcam, USA], Alix [ab186429, Abcam, USA], CD9 [ab263019, Abcam, USA]) and a negative marker (Calnexin [10427‐2‐AP, Proteintech]), were transferred onto a PVDF membrane. The membrane was then blocked with 3% BSA blocking solution, incubated with primary and secondary antibodies, and finally developed, fixed, and exposed for imaging.

### 2.7 The Olink Proteome Technique Method

Olink proteomics technology is an innovative protein detection and quantification method [28]. Olink Target 96 provides targeted protein biomarker panels designed to analyse specific proteins related to particular diseases or biological functions [29].

First, according to the manufacturer's instructions, total proteins are extracted from serum EVs to prepare protein samples. Antibodies conjugated with designed oligonucleotide sequences are used to bind to target proteins.

Afterwards, sample barcodes are added for library preparation, followed by qPCR analysis of the amplified sequences. The qPCR quantification data undergo preliminary processing via the NPX Signature software. Once imported into the Olink NPX Signature (NPX Manager) software, the data undergoes sample type annotation, protein NPX calculation, data quality control, and export. The final output is a Certificate of Analysis (CoA) and the NPX Data file, which serves as the input for subsequent bioinformatic analyses [30].

### 2.8 EVs Proteomics Comprehensive Analysis

Using the clean NPX data, we were able to conduct further analyses [31]. Differential protein expression analysis between the two groups (AAA group and healthy controls) was performed using a two‐tailed unpaired Student's *t*‐test. For multiple comparisons across multiple proteins, *p*‐values were adjusted using the Benjamini‐Hochberg method to control the false discovery rate (FDR). A protein was considered differentially expressed if it met the threshold of FDR‐adjusted *p* < 0.05 and absolute log2 fold change > 0.5. The results can be visualised through differential protein analysis, such as creating volcano plots and panel‐specific differential protein clustering heatmaps.

Further analysis of the differentially expressed proteins includes biological function enrichment analysis to explore the biological significance of the proteins in terms of Gene Ontology, KEGG Pathway, Reactome Pathway, and DOSE. Additionally, all proteins from the Olink project are annotated using relevant bioinformatics databases, followed by subcellular localisation analysis and protein–protein interaction network analysis [32].

### 2.9 Western Blot and ELISA Analysis for External Validation

Western Blot and enzyme‐linked immunosorbent assay (ELISA) were used to further validate the expression levels of five differentially expressed proteins (Oncostatin‐M, Interleukin‐4, Interleukin‐6, Neurturin, and MCP‐1) in an external cohort. The inclusion and exclusion criteria for the validation cohorts followed the standards outlined in Section 2.1. EVs were first isolated and purified using ultracentrifugation. Proteins were then extracted with RIPA Lysis and Extraction Buffer (Thermo #89900), followed by incubation with antibodies specific to IL‐4 (Proteintech #66142), IL‐6 (Proteintech #21865), MCP‐1 (Proteintech #26161), Neurturin (Proteintech #19709), and OSM (Proteintech #27792). The protein bands were visualised using ECL substrate after incubation with these antibodies. In a parallel process, proteins were extracted using RIPA buffer, with phosphatase inhibitors and PMSF added at a 100:1 ratio before use. The lysates were centrifuged at 13,000 *g* for 10 min at 4°C, and the supernatants were collected. Protein concentrations were measured using the BCA method, and these samples were used for ELISA detection. The ELISA kits utilised were: Human IL‐4 ELISA Kit (Multi Sciences, EK104/2‐96), Human IL‐6 ELISA Kit (Multi Sciences, EK106‐96), Human MCP‐1 ELISA Kit (Multi Sciences, EK187‐96), Human Neurturin ELISA Kit (YOBIBIO, U96‐1476E), and Human OSM ELISA Kit (YOBIBIO, U96‐1578E). Each kit was used according to the manufacturer's instructions.

### 2.10 Statistical Analysis

All statistical tests in this study were two‐tailed. A *p*‐value of < 0.05 and an FDR of < 0.05 were considered statistically significant. Descriptive statistics for continuous variables with normal distribution are reported as mean ± standard deviation. Comparisons involving continuous variables were conducted using the appropriate Wilcoxon rank‐sum test or Student's *t*‐test. Categorical variables were analysed using the chi‐square test or Fisher's exact test [20, 33]. All data processing, statistical analyses, and graphical plotting were performed using R software (version 4.4.3).

## 3 Results

### 3.1 Identification of Isolated Plasma EVs

**FIGURE 1:**
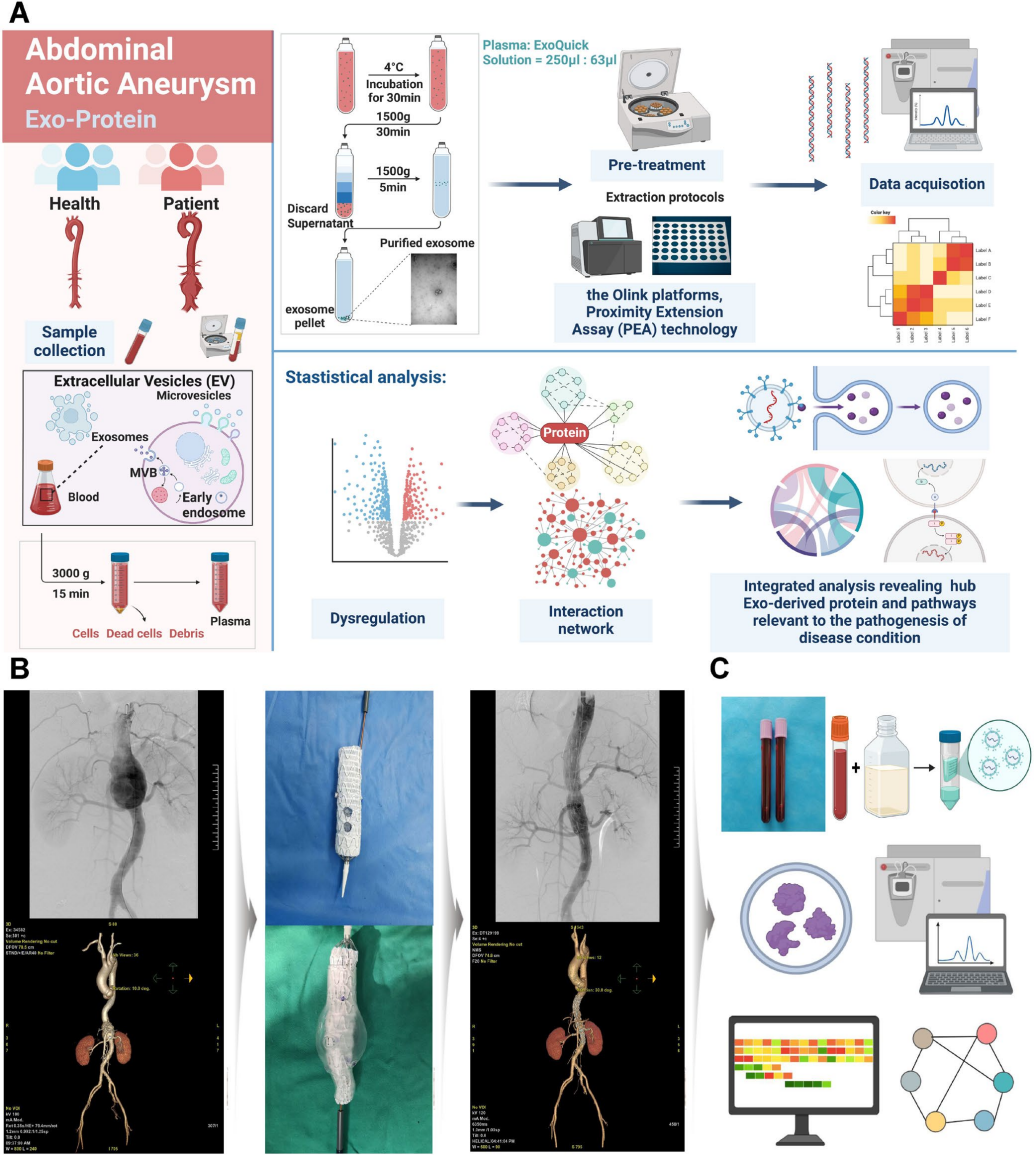
Extraction and processing workflow for circulating extracellular vesicles. (A) Complete workflow for high‐throughput sequencing and analysis of plasma exosome samples of AAA and normal people. (B) Pre‐ and postoperative aortic angiography, abdominal aorta CTA, and intraoperative stent customisation for patients with abdominal aortic aneurysm. (C) Blood collection from the abdominal aorta and the extraction and detection of circulating extracellular vesicles.

**FIGURE 2:**
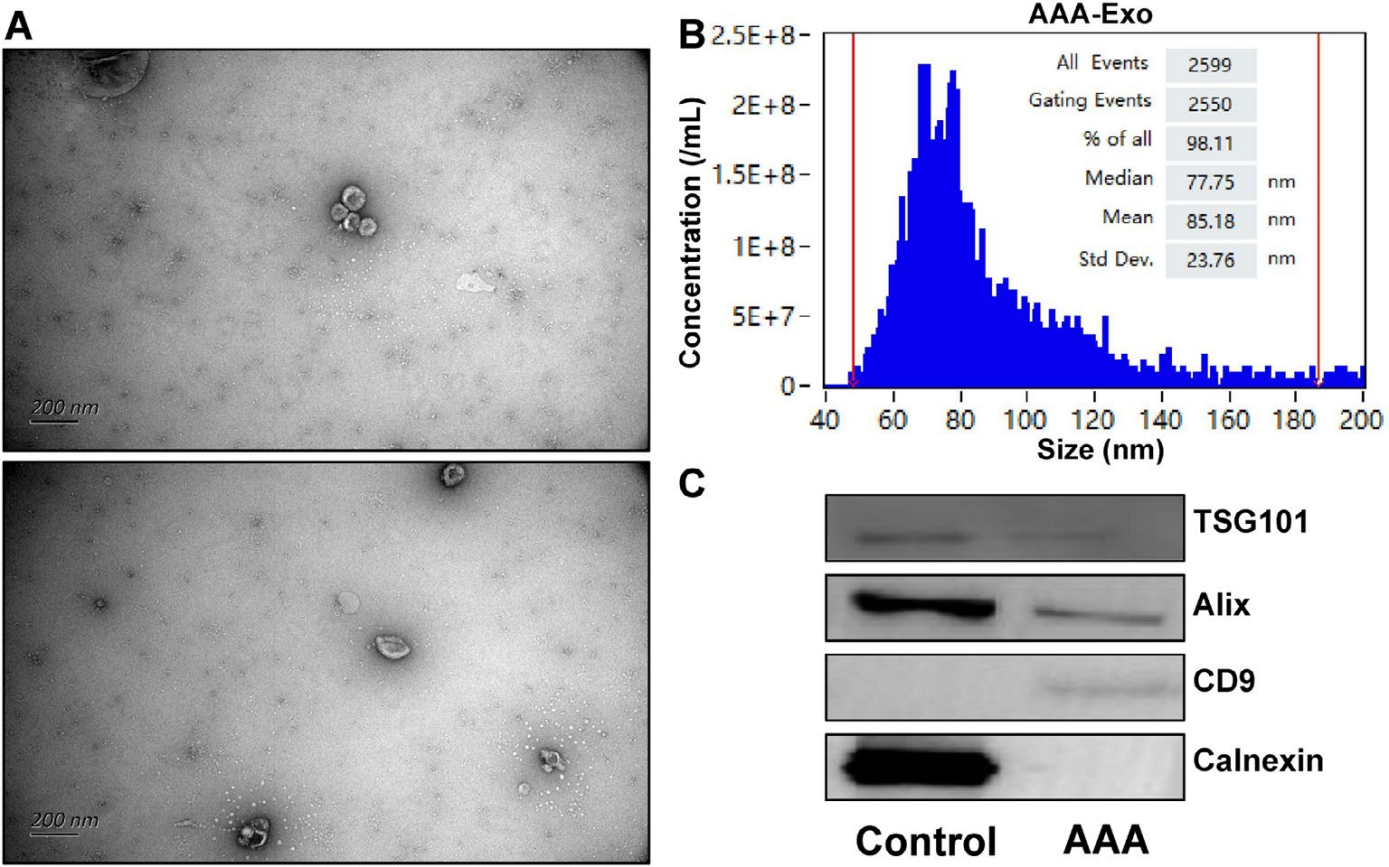
Characterisation and internalisation of AAA‐Exo. (A) Electron microscopic images of isolated EVs. Scale bar, 200 nm. (B) Results of high‐sensitivity flow cytometry for nanoparticle analysis of EVs. (C) Western blotting revealed Tsg101/Alix/CD9 proteins and EVs negative marker protein Calnexin in EVs and cell control samples.

The methodology of this study is summarised in an overall flowchart (Figure 1A). For AAA patients, a preoperative CTA assessment is conducted to reconstruct the abdominal aorta and create a 3D‐printed aneurysm model. Using the 3D aortic model, a customised stent is created with fenestrations based on the patient's anatomy. The stent is then positioned and deployed within the affected vessel. A final aortography is performed before closing to confirm complete exclusion of the aneurysm. A follow‐up CTA is performed one week post‐surgery (Figure 1B). During surgery, an aortography is first performed to confirm the lesion, followed by obtaining arterial blood directly from the patient's abdominal aorta before any further intervention; this blood sample is used for subsequent EVs extraction and analysis (Figure 1C). The characterisation of plasma‐derived EVs was based on their diameter, morphology, and surface protein expression. TEM images (Figure 2A) showed that the isolated plasma EVs exhibited the typical cup‐shaped structure. Nanoparticle analysis using a high‐sensitivity Flow NanoAnalyzer revealed that the median diameter of AAA EVs (AAA‐Exo) was 85.18 nm (Figure 2B). Additionally, Western blot analysis confirmed the expression of the protein markers Tsg101, Alix, and CD9, while no expression of Calnexin was detected in these EVs, indicating high purity and minimal cellular contamination of the EV's preparations (Figure 2C). Therefore, we successfully identified plasma EVs for subsequent protein biomarker detection.

### 3.2 Plasma EV Protein Biomarkers in AAA Patients Were Detected Using the Olink Proteome Technique

**FIGURE 3:**
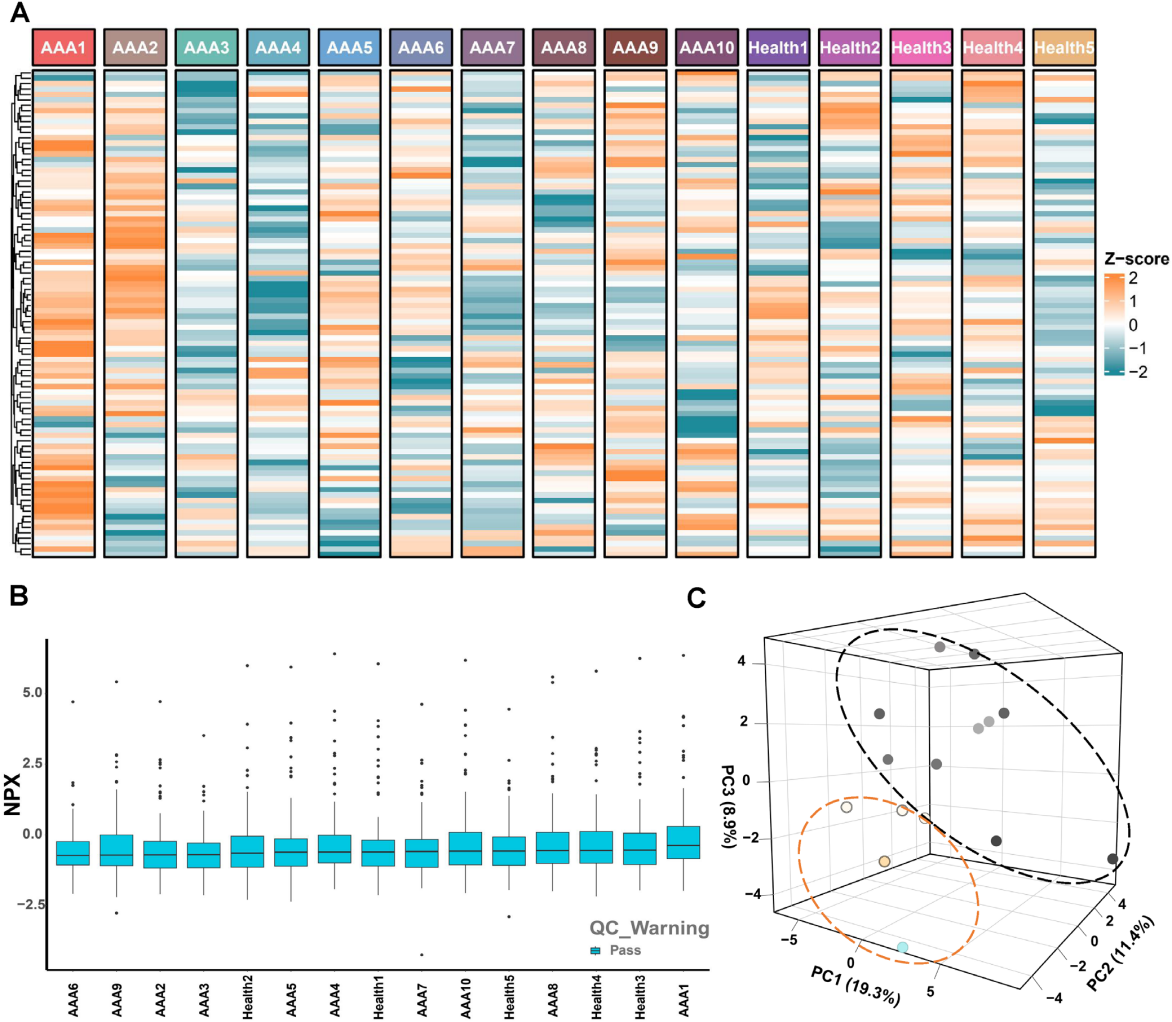
Circulating EV proteins expression. (A) Heatmap of all expressed plasma proteins between AAA and controls, where AAA represented the AAA group and Health represented the control normal group. (B) Box plot of NPX distribution of all expressed plasma proteins between AAA and controls, QC_Warning is labelled as a red Warning sample, Pass is a QC Pass sample and is labelled as a lime green Pass. (C) Principal component analysis plot of all expressed plasma proteins.

A total of 92 protein biomarkers were analysed in plasma EVs from AAA patients and healthy controls. The heatmap illustrates the landscape of EV protein biomarkers (Figure 3A). Boxplots show the NPX (Normalisation Protein expression) distribution of protein expression between the AAA group and healthy controls (Figure 3B). Principal component analysis (PCA) was performed to reduce dimensionality and visualise the complex sample data, with principal components representing the variation between samples (Figure 3C).

**FIGURE 4:**
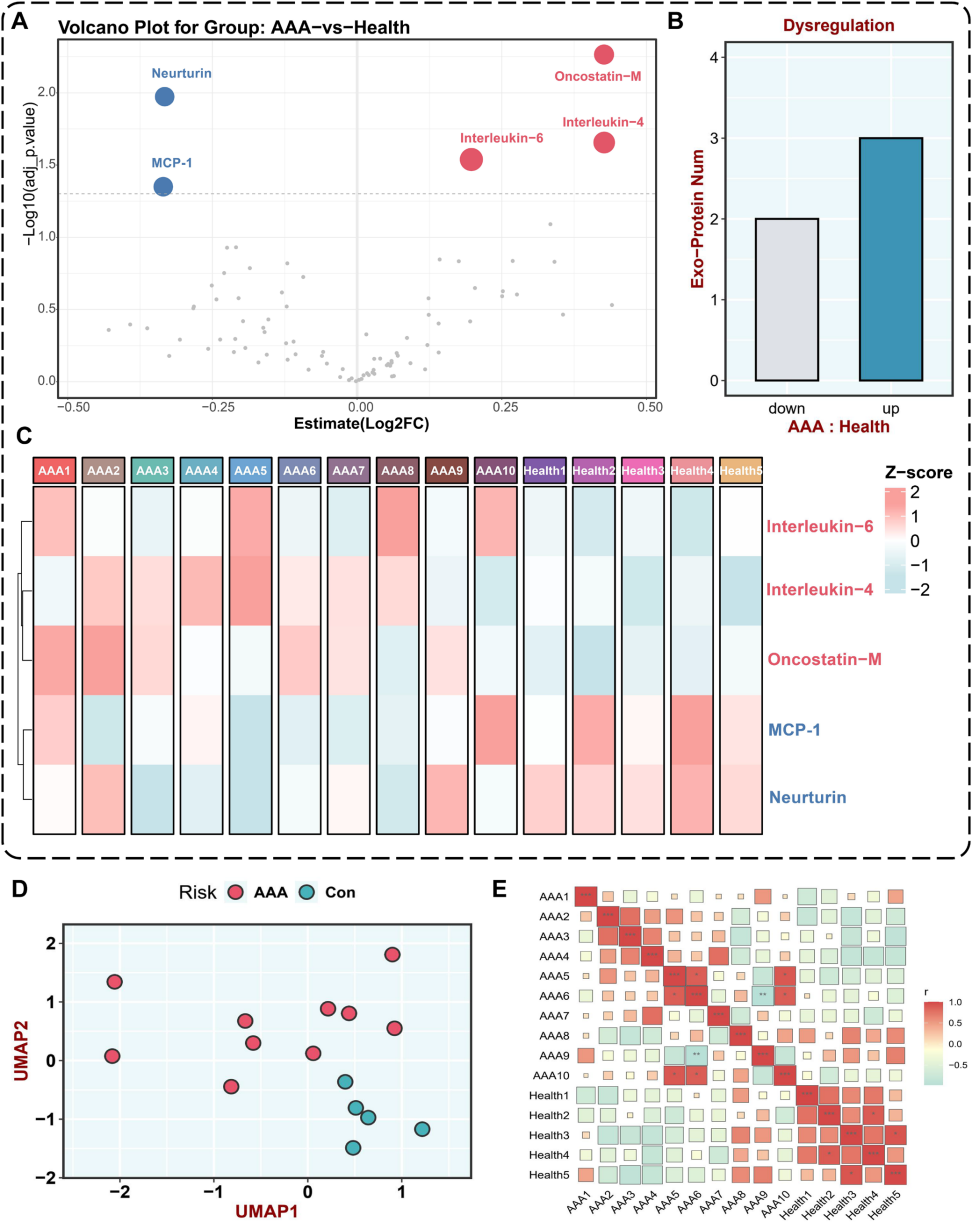
Clustering analysis of circulating EV differential proteins. (A) The volcano plot of plasma proteins. Red dots represent upregulated proteins; grey dots not‐significant proteins; and blue dots downregulated proteins. (B) Numbers of differentially expressed proteins (DEPs) in the two modules. (C) The heat map shows the 5 proteins expression profile under the threshold absolute logFC > 0.5 and *p* < 0.05, where AAA represented the AAA group and Health represented the control normal group. (D) Dimension reduction analysis of AAA and control samples for the plasma proteins expression profile. Dark red dots represent AAA samples; lime green dots represent control samples. (E) Pearson correlation analysis plot of normal samples and AAA samples.

At a significance threshold of *p* < 0.05, five protein biomarkers exhibited significant differential expression between the two groups, with three upregulated and two downregulated in the AAA patients' EVs. The downregulated protein biomarkers were further validated through volcano plots, bar charts, and differential protein clustering heatmaps (Figure 4A–C).

Additionally, Pearson correlation analysis and UMAP analysis were conducted to assess the similarities and differences in EV proteins expression between AAA patients and healthy controls (Figure 4D,E). These results reveal significant differences in the plasma EV proteins expression landscape between AAA patients and healthy individuals.

### 3.3 Comprehensive Functional Enrichment of Target Protein of AAA‐ EV‐Proteome

**FIGURE 5:**
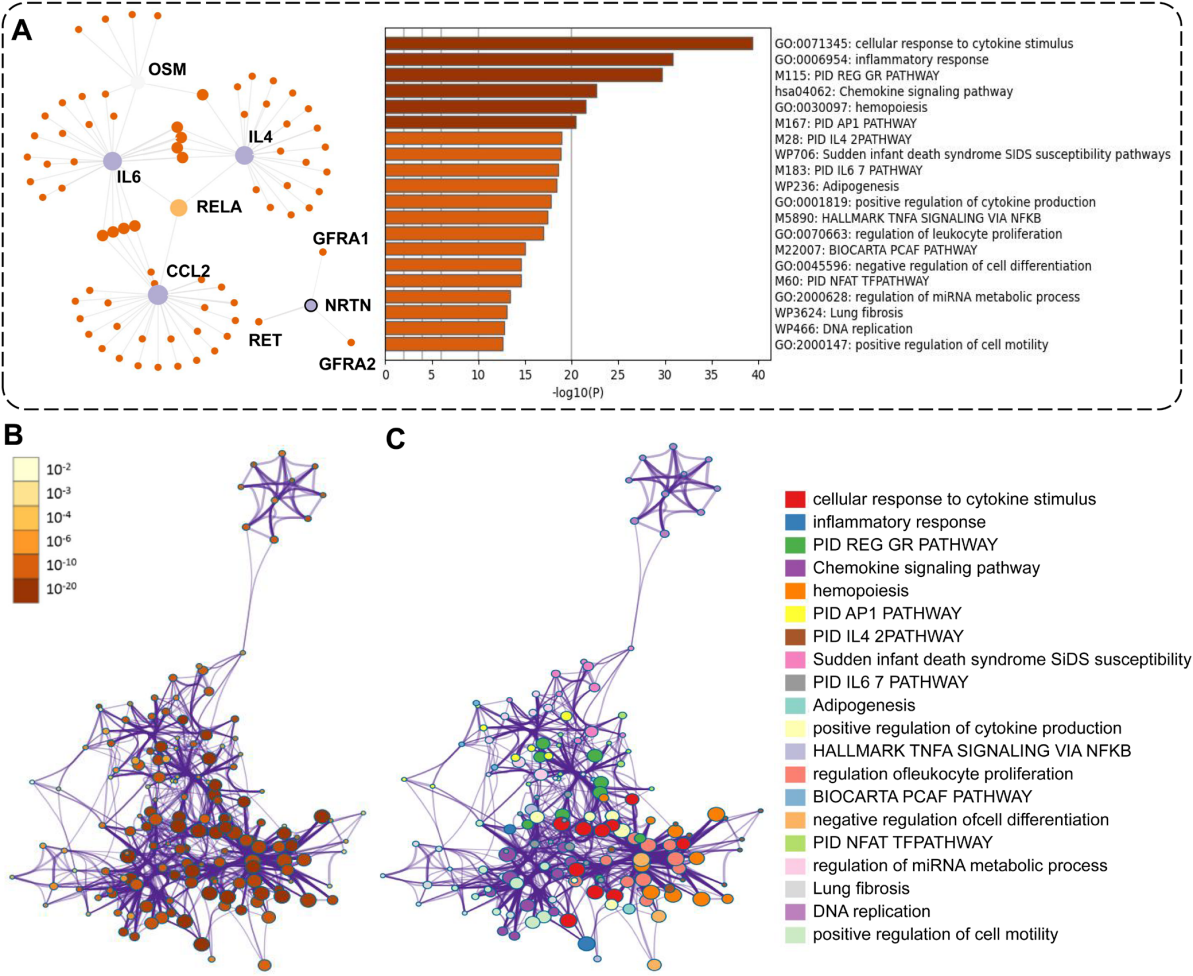
Biological implications of plasma differentially expressed proteins (DEPs) target genes. (A) The PPI network between the potential biomarkers was established by using the STRING database. The edge width represents the combined score. PPI, protein–protein interactions. Top 20 clusters from Metascape pathway and process enrichment analysis of proteins target genes, coloured by *p*‐values. (B and C) Network of enriched terms of DEPs target genes. Terms with a similarity > 0.3 are linked by edges: (B) coloured by enriched terms, where nodes that share the same enriched term are typically close to each other; (C) coloured by *p*‐value, where terms containing more genes tend to have a more significant *p*‐value.

We annotated all identified AAA‐EV proteins using the STRING database and conducted a protein–protein interaction (PPI) analysis. This resulted in PPI interaction networks labelled with both UniProt IDs and Gene Symbols. After removing isolated target genes lacking interactions and applying a confidence threshold of 900, we visualised the gene interaction network using Cytoscape (Figure 5A). The top 20 enriched terms of key clusters from this analysis are displayed in the figure. To further clarify the relationships among these top terms, we constructed a functional annotation network, as shown in Figure 5B,C. This network provides deeper insights into the potential interconnected pathways and processes influenced by AAA‐EV‐derived proteins, enhancing our understanding of their roles in the molecular mechanisms of AAA.

After the initial analysis, we conducted a comprehensive landscape and process enrichment analysis on the predicted targets of AAA‐EV‐proteins. This analysis integrated data from multiple ontological sources, including GO Biological Processes, GO Molecular Functions, KEGG Pathways, Human Gene Set, Reactome Gene Set, and canonical pathways. The results highlighted several significantly enriched terms, notably “cellular response to cytokine stimulus” (GO:0071345), “inflammatory response” (GO:0006954), Chemokine signalling pathway (hsa04062), the activator protein‐1 (AP‐1) pathway (M167) and the regulation of glucocorticoid receptor (GR) pathway (M115). Previous studies have found that inflammatory responses, along with the production and activation of various proteases, play a crucial role in promoting the formation and progression of aortic aneurysms.

### 3.4 Western Blot and ELISA Validation of Differentially Expressed Proteins in Circulating Extracellular Vesicles of AAA

Five proteins that were significantly dysregulated between AAA and healthy individuals were identified as hub proteins (IL‐4, IL‐6, MCP‐1, Neurturin, and Oncostatin‐M). Next, we validated the manifestation of hub proteins in an external population (circulating EVs from 8 patients and 4 healthy controls) based on Western Blot and ELISA.

**FIGURE 6:**
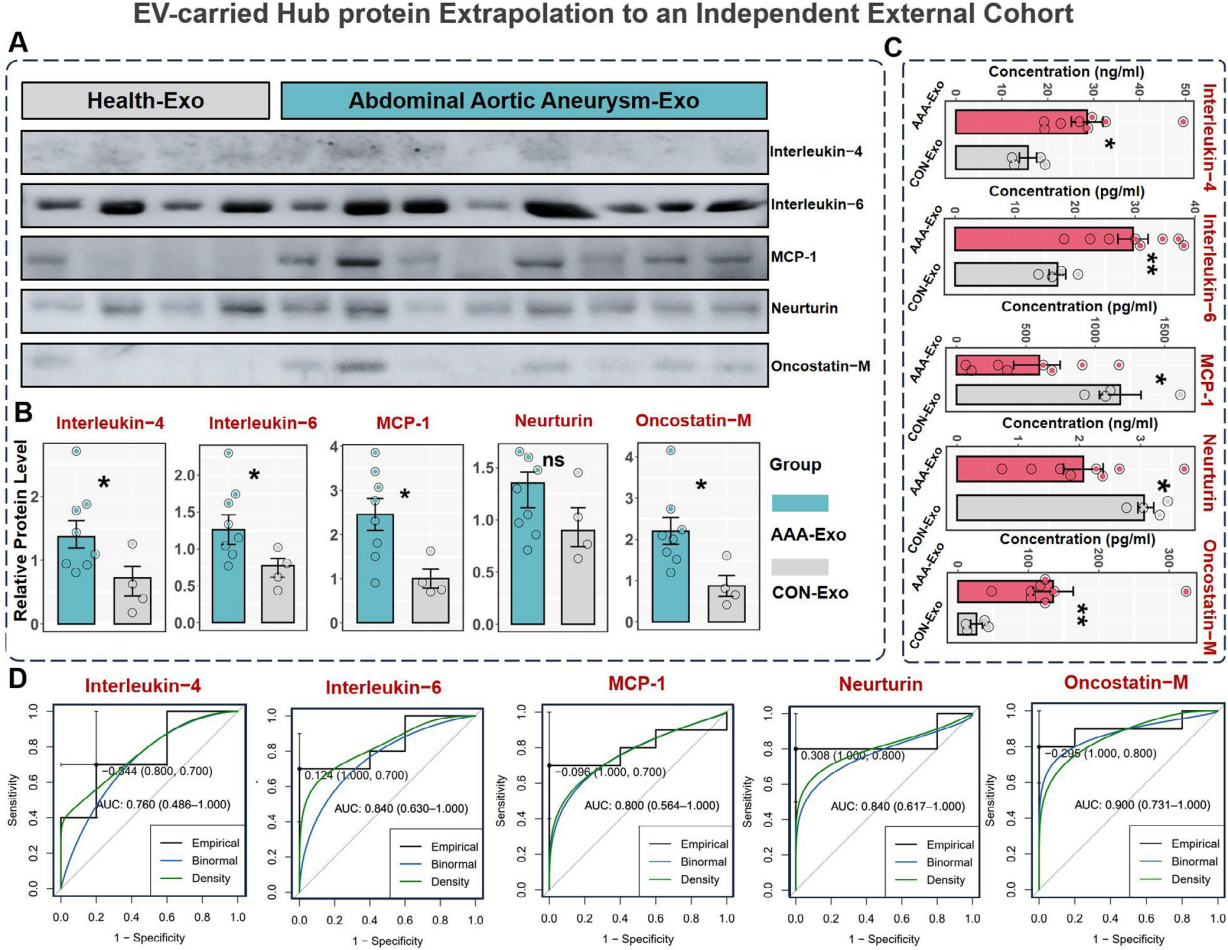
EV‐carried Hub protein Extrapolation to an Independent External Cohort. (A) Western blotting revealed plasma proteins of IL‐4, IL‐6, MCP‐1, Neurturin, and Oncostatin−M. Health‐Exo = 4, AAA‐Exo = 8. (B) The distribution of 5 hub protein expression level between AAA and healthy control groups in the external cohort based on Western blotting strip grayscale analysis. *p < 0.05 (Wilcoxon test) and ns represents no significance. (C) Elisa validation of plasma IL‐4, IL‐6, MCP‐1, Neurturin, and Oncostatin−M, * represent data compared with CON, *p < 0.05, **p < 0.01. AAA‐Exo is labelled as a red sample, Health‐Exo is labelled as a grey sample. (D) Elisa‐validated ROC curves for core proteins from external cohorts. (Due to the limited sample size, some proteins exhibited wide AUC confidence intervals. Further validation in larger cohorts is warranted.)

Following the Western Blot imaging of the five differentially expressed proteins, the developed bands are shown in Figure 6A. Grayscale analysis of the bands indicated that Interleukin‐4, Interleukin‐6, MCP‐1, and Oncostatin‐M were significantly elevated in AAA patients compared to the control group (*p* < 0.05), while Neurturin showed no statistically significant difference (Figure 6B). Subsequently, these five proteins were further validated using ELISA. The ELISA results (Figure 6C) demonstrated that circulating levels of interleukin‐4, interleukin‐6, and tumour suppressor‐M were significantly higher in AAA‐Exo samples, while MCP‐1 and Neurturin levels were significantly lower compared to healthy controls (*p* < 0.05). Additionally, Figure 6D displays the AUC‐ROC curves for these five proteins from the ELISA results, with AUC values of 0.760 (95% CI: 0.486–1.000), 0.840 (95% CI: 0.630–1.000), 0.800 (95% CI: 0.564–1.000), 0.840 (95% CI: 0.617–1.000), and 0.900 (95% CI: 0.731–1.000), respectively. These AUC values indicate strong diagnostic potential, suggesting that these differentially expressed proteins possess a robust ability to distinguish AAA patients from healthy individuals.

## 4 Discussion

AAA is a disease with a subtle onset; patients often remain asymptomatic until the aneurysm ruptures, a complication with a mortality rate as high as 75%–90%. Given the long latency between the onset of AAA and the need for treatment, identifying a biomarker for screening or monitoring disease progression would facilitate the development of cost‐effective blood‐based screening methods. From a clinical perspective, the EV‐associated proteins identified in our study hold promise as minimally invasive biomarkers for AAA. Currently, AAA diagnosis and monitoring rely primarily on imaging modalities (such as ultrasound or CT scans) that measure aneurysm size, which does not always reflect the underlying biological activity or rupture risk of the aneurysm. Circulating EV protein biomarkers could complement imaging by providing molecular insight into disease activity. For instance, elevated levels of inflammatory EV proteins (like IL‐6 or MCP‐1) might indicate active aneurysm wall inflammation or expansion even when the aneurysm is below the typical intervention size [34]. If validated in larger cohorts, an EV‐based biomarker panel could be used alongside imaging to improve early detection, risk stratification, and monitoring of AAA patients. Integrating such blood‐based biomarkers with standard imaging could thus lead to a more comprehensive and personalised approach to AAA management, potentially enabling earlier interventions and better outcomes.

In this study, we describe specific protein biomarkers in the plasma EVs of AAA patients. Using Olink's PEA technology, we quantitatively analysed proteins in the EVs of AAA patients. Our analysis revealed differentially expressed EV protein biomarkers, including three upregulated and two downregulated biomarkers. Through enrichment analyses, we predicted the regulatory functions of these protein biomarkers in AAA. These analyses highlighted key pathways, particularly focusing on “cellular response to cytokine stimulus”, “the regulation of glucocorticoid receptor pathway,” “Chemokine signaling pathway,” and “the activator protein‐1 pathway.” This study elucidates the molecular mechanisms linking AAA to plasma EV proteins and proposes potential therapeutic targets.

The “cellular response to cytokine stimulus” is a critical biological process involving a series of cellular responses to cytokine signals. They are associated with various diseases, including cardiovascular diseases. The formation and progression of AAA are closely related to localised chronic inflammation. Pro‐inflammatory cytokines (such as TNF, IL‐1, IL‐6) can activate smooth muscle cells, endothelial cells, and macrophages, leading to the upregulation of MMPs and degradation of the arterial wall [6, 35]. Some cytokines (such as TNF) induce apoptosis in smooth muscle cells and other cells through their signalling pathways, further compromising the structural integrity of the arterial wall [36]. Therefore, modulating the interaction between anti‐inflammatory and pro‐inflammatory factors to alleviate arterial wall degradation may become a potential target for AAA treatment.

The regulation of the GR pathway plays a crucial role in regulating immune responses, inflammatory responses, and cellular metabolism. The GR binds to glucocorticoids such as cortisol, inhibiting the expression of pro‐inflammatory cytokines like IL‐6 and TNF‐α [37]. The AP‐1 signalling pathway is an important transcription factor pathway that plays a key role in regulating cell proliferation, differentiation, apoptosis, and stress responses. AP‐1 can also regulate the expression of chemokines, attracting immune cells to the arterial wall and promoting localised inflammation. These pro‐inflammatory factors are critical in the development of AAA, leading to the degradation and expansion of the arterial wall through the promotion of inflammatory cell infiltration and activation [38]. De Bosscher et al. found that GR reduces the expression of pro‐inflammatory cytokines by inhibiting the activity of transcription factors such as NF‐κB and AP‐1. MMPs (e.g., MMP‐2, MMP‐9) are highly expressed in AAA, leading to the degradation and remodelling of the arterial wall matrix. Eberhardt et al. also found that activation of the GR can indirectly reduce MMP expression by inhibiting the activity of transcription factors like NF‐κB and AP‐1 [39].

The Chemokine Signalling Pathway is an essential biological pathway involved in various physiological and pathological processes such as cell migration, immune responses, and inflammation. Chemokines bind to G protein‐coupled receptors (GPCRs) on the cell membrane, activating the receptors. This activation induces conformational changes in the associated G proteins, triggering a signalling cascade that can activate various downstream pathways such as Ras‐MAPK, PI3K‐Akt, and JAK–STAT [40, 41, 42]. Zhang et al. [43] suggested that pharmacological blockade of CCR2 and its antagonists could affect the infiltration of mast cells and other inflammatory cells into human AAA lesions, potentially preventing or reducing AAA formation or progression. Our study also reveals the critical role of the Chemokine Signalling Pathway in the EV protein markers associated with AAA.

However, our study has limitations. The small sample size and specific patient population we studied limit the generalisability of our findings. For instance, we imposed strict inclusion criteria (including a restricted BMI range of 20–35 and exclusion of certain comorbidities) to reduce confounding factors; while this homogeneity strengthens internal validity, it may reduce applicability to the broader AAA patient population. Moreover, we observed partial inconsistencies between Western blot and ELISA results, particularly in the case of Neurturin, where expression trends differed between the two assays. This discrepancy may be attributed to several factors, including variations in sample preparation, differences in the sensitivity and specificity of detection methods, or biological heterogeneity among the small number of samples used. Given the limited sample size and the technical variability inherent in protein quantification platforms, such inconsistencies are not uncommon and underscore the need for validation in larger, independent cohorts using multiple orthogonal detection methods. Additionally, circulating EVs can originate from many tissues. We cannot be certain that the EVs we analysed were released specifically from aneurysm tissue. Further experiments using human aneurysm tissue (e.g., isolating EVs directly from surgically resected AAA tissue or using cell‐specific EV markers) would be valuable to confirm that these proteins indeed derive from the aneurysmal aorta and to validate our bioinformatic predictions in the future.

Furthermore, although our analysis revealed significant associations between key EV‐derived proteins (such as IL‐6, MCP‐1, and Oncostatin‐M) and AAA‐related inflammatory signalling pathways (e.g., AP‐1, GR, and chemokine signalling), the current findings remain at the correlative level due to the lack of direct functional validation. We did not perform functional experiments (e.g., gene knockdown or overexpression) in this study to directly test the roles of these proteins. Therefore, no causal inferences can be drawn at this stage. To advance from association to mechanistic insight, future studies are necessary to employ in vitro and in vivo models—such as gene silencing, overexpression, or CRISPR‐based perturbation of candidate EV proteins—to determine their direct impact on vascular smooth muscle cell behaviour, matrix remodelling, and aneurysm progression. For instance, IL‐6 and MCP‐1 have previously been shown to promote aneurysm formation by enhancing macrophage infiltration and MMP activation [44, 45]; thus, targeting these molecules in animal or organoid models may validate their pathogenic roles.

Notwithstanding these limitations, our proteomic findings are supported by prior studies of AAA. For example, IL‐6 and MCP‐1, which we found to be elevated in EVs from AAA patients, are well‐known contributors to AAA pathogenesis by promoting inflammatory cell recruitment and matrix degradation. Oncostatin‐M, an IL‐6 family cytokine that we identified as upregulated in AAA EVs, has been shown to induce inflammatory pathways in vascular tissues, suggesting it could similarly exacerbate aneurysm development. By contrast, IL‐4 is typically anti‐inflammatory, and its reduced level in AAA EVs may reflect the shift toward a pro‐inflammatory environment within the aneurysm. Neurturin, a neurotrophic factor, has not been widely studied in AAA and thus represents a novel finding of this study, warranting further investigation. Future functional experiments focusing on these molecules will be crucial to determine their exact contributions to AAA progression and to validate them as potential therapeutic targets.

## Author Contributions

**Chaoyang Yu:** conceptualization (equal), formal analysis (equal), investigation (equal), methodology (equal), software (equal), visualization (equal), writing – original draft (equal). **Ge Zhang:** conceptualization (equal), formal analysis (equal), methodology (equal), software (equal), visualization (equal), writing – review and editing (equal). **Shaotong Pei:** conceptualization (equal), formal analysis (equal), investigation (equal), software (equal), visualization (equal). **Yifei Zhang:** investigation (equal), validation (equal), visualization (equal). **Peiyu Yuan:** investigation (equal), software (equal), validation (equal). **Renying Miao:** investigation (equal), resources (equal), visualization (equal). **Kaisaierjiang Kadier:** investigation (equal), validation (equal), writing – review and editing (equal). **Pengpeng Zhang:** investigation (equal), validation (equal), writing – review and editing (equal). **Tianshu Gu:** investigation (equal), validation (equal), writing – review and editing (equal). **Ruhao Wu:** investigation (equal), validation (equal). **Haonan Zhang:** investigation (equal), validation (equal). **Shiqian Zhang:** investigation (equal), visualization (equal). **Bo Yang:** investigation (equal), visualization (equal). **Han Wu:** investigation (equal), validation (equal). **Yudi Xu:** investigation (equal), validation (equal). **Ke Hu:** investigation (equal), visualization (equal). **Qingfei Xu:** investigation (equal), validation (equal). **Yaxin Chen:** investigation (equal), validation (equal). **Jinliang Wang:** investigation (equal), validation (equal). **Zongao Cai:** investigation (equal), visualization (equal). **Junnan Tang:** conceptualization (equal), methodology (equal), writing – review and editing (equal). **Yan Song:** conceptualization (equal), funding acquisition (equal), methodology (equal), project administration (equal), resources (equal), writing – review and editing (equal). **Teng Li:** conceptualization (equal), methodology (equal).

## Ethics Statement

This study was conducted according to the ethical guidelines of the Declaration of Helsinki and approved by the Institutional Review Board of the First Affiliated Hospital of Zhengzhou University (Approval Number: 2024‐KY‐0095).

## Consent

Prior informed written consent for specimen collection and subsequent analysis was secured from each participant.

## Conflicts of Interest

The authors declare no conflicts of interest.

## Data Availability

Individual protein data for this study were obtained from the electronic health record system of the Department of Vascular and Endovascular Surgery, the First Affiliated Hospital of Zhengzhou University in Henan Province. Due to data privacy laws, ethical restrictions, and confidentiality agreements, internal data at the individual level are protected from public sharing. For additional information to reanalyse the data supporting the findings, please contact the corresponding author (fccsongy3@zzu.edu.cn) with a detailed request, which may require a signed data use agreement to ensure subject confidentiality. The Ethics Committee of the First Affiliated Hospital of Zhengzhou University in Henan Province will evaluate the application and respond within 30 working days.
